# Comparison of complications of intrascleral fixation according to the extent of vitrectomy

**DOI:** 10.1186/s12886-024-03430-9

**Published:** 2024-04-09

**Authors:** Miho Yamada, Eiichi Nishimura, Sayako Watanabe, Masanori Yoshino, Yoshiro Tokunaga, Natsuko Sugiyama, Mitsutaka Soda

**Affiliations:** Department of Ophthalmology, Showa University Fujigaoka Rehabilitation Hospital Aobaku Yokohamashi, Kanagawa 2-1-1, Japan

**Keywords:** Vitrectomy, Intrascleral fixation, Intraocular lens

## Abstract

**Background:**

Intraocular lens (IOL) fixation is performed after intraoperative anterior or total vitrectomy. This study aimed to compare the intraoperative and postoperative complications of these two techniques.

**Methods:**

This retrospective study included 235 eyes that underwent intrascleral fixation surgery at our hospital between July 2014 and January 2021. The eyes were classified into the anterior vitrectomy group (A-vit group; 134 eyes) and the pars plana vitrectomy group (PPV group; 101 eyes). The age, preoperative and postoperative best-corrected visual acuity, observation period, preoperative and postoperative intraocular pressure, and the incidence of intraoperative and postoperative complications were assessed.

**Results:**

Intrascleral fixation was performed more frequently in the PPV group, and a significant difference was observed between the eyes with a history of vitrectomy and eyes with scleral buckles (*p* = 0.00041). In terms of the incidence of postoperative complications following intrascleral fixation, the incidence of low intraocular pressure postoperative was higher in the PPV group than that in the A-vit group, and a significant difference was observed between the two groups (*p* = 0.01).

**Conclusions:**

The visual outcome and complications following intrascleral fixation did not differ according to the extent of vitreous excision.

**Supplementary Information:**

The online version contains supplementary material available at 10.1186/s12886-024-03430-9.

## Background

Intrascleral fixation of the intraocular lens (IOL) was first reported by Gabor et al. in 2007 [1]. Kobayakawa et al. introduced this technique in Japan in 2010, and this technique has garnered attention as a method for IOL fixation in patients with aphakic lens [2–4]. To perform intrascleral fixation successfully, the support of the IOL inserted intraocularly must be pulled out, the IOL must be inserted into the sclera, and fixed in place. Intraoperative anterior or total vitrectomy must be performed prior to this technique. However, no previous reports have compared these two techniques. Therefore, this study compared the intraoperative and postoperative complications of these two techniques.

## Methods

This study included 235 eyes that underwent intrascleral fixation at the Showa University Fujigaoka Rehabilitation Hospital between July 2014 and January 2021. The eyes were classified into the anterior vitrectomy group (A-vit group; 134 eyes) and the pars plana vitrectomy group (PPV group; 101 eyes) according to the extent of vitreous excision performed at the time of intrascleral fixation. The A-Vit group is basically used for subluxation cases, while the PPV group is used for cases in which the IOL is dislocated and falls into the vitreous body, and even the surrounding vitreous body is resected. The age, observation period, preoperative and postoperative best-corrected visual acuity, preoperative and postoperative intraocular pressure (within 7 postoperative days), and incidence of intraoperative and postoperative complications were compared between the two groups. Statistical analysis was performed using a two-sample t-test and chi-square test with unequal variances. t-test: two-sample test assuming equal variances. After confirming the normal distribution, the corresponding t-test was used to confirm the normal distribution. In all examinations, a statistical significance level of 0.05 was used for the t-tests.

Landolt rings (C mark) are used in all vision tests.

The intrascleral fixation techniques used in this study were the Y-fixation and T-fixation with forceps technique [4, 5] or Yamane’s method with double-needle and flange technique [6–8]. The extent of vitrectomy was determined according to the location of the dislocated lens or IOL and the location of the fallen nucleus and cortex. Anterior vitrectomy was performed if the lens or IOL could be visualized from the pupillary field and the anterior approach was possible. Trans-ciliary vitrectomy was performed if the anterior approach could not be used or the lens, nuclear cortex, and IOL were dislocated into the posterior vitreous. An infusion port was placed in the corneal limbus or a pars plana in the A-vit group. Subsequently, the dislocated lens, nuclear cortex, and IOL were removed, and the prolapsed vitreous was visualized after the administration of triamcinolone acetonide (McQuaid^®^, Wakamoto Pharmaceuticals). Vitrectomy was performed in the anterior chamber and vitreous after the excision of the prolapsed vitreous. Acetylcholine (ovisort^®^) was injected into the anterior chamber to constrict the intraocular lens prior to its intrascleral fixation, and the remaining vitreous on the iris was wiped with a push-and-pull hook. The surgery was concluded with a core-vitrectomy to confirm that there was no vitreous traction. Three to four ports were created in the pars planar area in the pars plana vitrectomy group (PPV group). First, central vitrectomy was performed, and the vitreous was visualized using McQuaid^®^ to evaluate for the presence of posterior vitreous detachment. Posterior vitreous detachment was artificially created if it was not detected. The visible vitreous was excised using a wide-angle viewing system, and the dislocated lens, nuclear cortex, or IOL were removed. Intrascleral fixation was performed after checking for retinal tears and detachment to avoid intraoperative trouble.The loop of the IOL was fixed symmetrically at approximately 2 mm from the corneal limbus during intrascleral fixation. In the forceps method, the tip of the IOL loop (support part) was pulled out of the eye and inserted into the scleral tunnel, and the IOL loop was fixed to the scleral bed with a single suture. In the double-needle method, the support was pulled out of the eye, the length of the tip was adjusted, and the flange method was performed subsequently. Peri-iridectomy was performed in all cases to prevent the development of reverse pupil block, and the operation was terminated with removal of the infusion cannula. The incisions for IOL removal and trocar insertion were sutured subsequently; the surgery was completed with 8 − 0 Bicryl^®^ ( Johnson & Johnson) without sutures if there was no leakage. The 25-gauge Constellation^®^ (Alcon Laboratories, Fort Worth, USA) and wide-angle viewing system (Resight^®^, Carl Zeiss Meditec, Dublin, USA) were utilized for vitrectomy and observation, respectively, in both groups. Both groups are to check the fundus.

This study was performed in accordance with the tenets of the Declaration of Helsinki and its later amendments and was approved by the Institutional Review Board/Ethics Committee of the Showa University Fujigaoka Rehabilitation Hospital. Approval number F2020C62.Written informed consent was obtained from the patient for the surgery and study. Consent is obtained at the same time as the surgical consent form.

## Results

The A-vit and PPV groups comprised 134 and 101 eyes, respectively. There were 92 men and 42 women in the A-vit group and 71 men and 30 women in the PPV group (*p* = 0.90). The mean age of the participants in the A-vit and PPV groups was 67 ± 15.8 and 68 ± 15.1, respectively (*p* = 0.50). The preoperative visual acuity (logMAR) in the A-vit and PPV groups was 0.353 and 0.325, respectively (*p* = 0.48). The postoperative best-corrected visual acuity (logMAR) in the A-vit and PPV groups was 0.112 and 0.112, respectively (*p* = 0.59).

The mean observation period in the A-vit and PPV groups was 25.5 ± 21.0 months and 24.3 ± 17.2 months, respectively (*p* = 0.64) (Table 1) There was a significant difference in the duration of follow-up between patients who could be followed up for a long time and those who were unable to go to the hospital or did not visit the hospital.; no significant difference was observed between the groups. The most common cause of intrascleral fixation in the PPV group was previous vitrectomy or scleral buckle (nine and 24 eyes in the A-vit and PPV groups, respectively; *p* = 0.00041). Trauma was the most common cause of intrascleral fixation in the A-vit group (19 and 10 eyes in the A-vit and PPV groups, respectively; *p* = 0.43). Intraoperative damage to the ciliary zonule was observed in 16 and 15 eyes in the A-vit and PPV groups, respectively (*p* = 0.65). Pseudoexfoliation syndrome was observed in 17 eyes and eight eyes in the A-vit and PPV groups, respectively (*p* = 0.34). Complications related to atopic dermatitis were observed in 13 eyes and nine eyes in the A-vit and PPV groups, respectively (*p* = 0.98). Glaucoma was observed in eight eyes each in the A-vit and PPV groups (*p* = 0.74). Intraoperative posterior capsule rupture (PCR) was observed in 6 and 1 eyes in the A-vit and PPV groups, respectively (*p* = 0.24), and Marfan syndrome was observed in 2 and 0 eyes in the A-vit and PPV groups, respectively (*p* = 0.61). The most common cause of intrascleral fixation was previous vitrectomy and scleral buckle in the Vit group, and a significant difference was observed between the A-vit and PPV groups (*p* = 0.00041). (Table 2)

**Table 1:** Research Background

	Number of cases	Gender Male/Female	Mean age	Preoperative visual acuity(logMAR)	Postoperative visual acuity(logMAR)	Observation period
A-vit group	134	92/42	67 ± 15.8	0.353	0.112	25.5 ± 21.0 months
Vit group	101	71/30	68 ± 15.1	0.325	0.112	24.3 ± 17.2 months
p-value		0.9	0.5	0.48	0.59	0.64

**Table 2:** Causes of intrascleral fixation

	Post vitrectomy and post scleral buckling	Trauma	Intraoperative damage to the ciliary zonule	Pseudoexfoliation Syndrome	Atopic	Glaucoma attack	PCR	Marfan syndrome	Cause unkown
A-vit group	9	19	16	17	13	8	6	2	44
Vit group	24	10	15	8	9	8	1	0	26
p-value	0.00041	0.43	0.65	0.34	0.98	0.74	0.24	0.61	

PCR, posterior capsule breakage

The following intraoperative complications of intrascleral fixation were observed: iris damage in two eyes (1.5%) and two eyes (2.0%) in the A-Vit and PPV group, respectively (*p* = 0.82); IOL drop in one (0.75%) and two (2.0%) eyes in the A-Vit and PPV group, respectively (*p* = 0.80); and choroidal hemorrhage in one (0.75%) and zero eyes in the A-Vit and PPV group (*p* = 0.89), respectively. Retinal tear formation did not occur in either group. The incidence of intraoperative complications was < 2% in both groups and no significant difference was observed between the groups. No serious complications were observed in either group (Table 3).

**Table 3:** Intraoperative complications of intrascleral fixation

	Iris rupture	IOL drop	Choroidal hemorrhage
A-vit group	2 (1.5%)	1 (0.75%)	1
Vit group	2 (2.0%)	2	0
p-value	0.82	0.8	0.89

IOL, intraocular lens

The postoperative complications included high postoperative intraocular pressure in 14 (10.4%) and 13 (12.9%) eyes in the A-vit and PPV groups, respectively (*p* = 0.71); low postoperative intraocular pressure in 11 (8.2%) and 21 (20.8%) eyes in the A-vit and PPV groups, respectively (*p* = 0.01) Low IOP was defined as 6.5 mmHg or less, High IOP was defined as 21 mmHg or higher.; macular edema in three (2.2%) and six (5.9%) eyes in the A-vit and PPV groups, respectively (*p* = 0.26); pars plana detachment in three (2.2%) and two (2.0%) in the A-vit and PPV groups, respectively (*p* = 0.75); and subconjunctival exposure of the loop of IOL in four (3.0%) and two (2.0%) eyes in the A-vit and PPV groups, respectively (*p* = 0.95) (Table 4). Pars plana detachment was diagnosed by CASIA2 Advance TOMEY.

**Table 4:** Postoperative complications of intrascleral fixation (1)

	High postoperative intraocular pressure	Low postoperative intraocular pressure	Macular edema	Ciliary detachment	Subconjunctival exposure of the loop of IOL
A-vit group	14	11	3	3	4
	(10.4%)	(8.2%)	(2.2%)		(3.0%)
Vit group	13	21	6	2	2
	(12.9%)	(20.8%)	(5.9%)	(2.0%)	
p-value	0.71	0.01	0.26	0.75	0.95

IOL, intraocular lens

The severity of low postoperative intraocular pressure was significantly greater in the PPV group than that in the A-vit group (*p* = 0.01). Iris capture was observed in three eyes (3.0%) in the PPV group (*p* = 0.16), retinal detachment was observed in two eyes (1.5%) in the A-vit group (*p* = 0.61), IOL tilt was observed in two eyes (1.5%) in the A-vit group and one eye (1.0%) in the PPV group (*p* = 0.80), and breakage of the loop was observed in one eye in the A-vit group (*p* = 0.89). Postoperative developed epiretinal membrane (ERM) was observed in two eyes (1.5%) in the A-vit group (*p* = 0.61), and a macular hole was observed in one eye (0.75%) in the A-vit group and one eye (1.0%) in the PPV group (*p* = 0.61) during the postoperative follow-up. No significant differences were observed between the two groups. Postoperative endophthalmitis, which was likely elicited by a corneal wound infection, was observed on the 22nd postoperative day in one eye (0.75%) with a history of atopic dermatitis in the A-vit group (*p* = 0.89). Emergency vitrectomy was performed on the same day, and the IOL was preserved. Eyes were circulated through the vitreous with a 0.125% balanced salt solution containing ceftazidime (Modacin®) and vancomycin. The results of intraoperative bacterial culture for the anterior chamber and vitreous were negative. The postoperative course was good, and the logMAR visual acuity was maintained at − 0.08. Vitreous hemorrhage was not observed in either group that required procedure after surgery. Vitreous hemorrhage itself was not observed (Table 5).

**Table 5:** Postoperative complications of intrascleral fixation (2)

	IOL Iris capture	Retinal detachment	IOL slant	Damage to the support section	ERM	MH	Endophthalmitis
A-vit group	0	2	2	0	2	1 (0.75%)	1
		(1.5%)					
Vit group	3	0	1	1	0	1	0
	(3.0%)		(1.0%)				
p-value	0.16	0.61	0.8	0.89	0.61	0.61	0.89

IOL, intraocular lens; ERM, epiretinal membrane; MH, macular hole

The average duration of surgery was 44 and 71 min for A-vit and PPV groups, respectively (*p* = 1.06). No significant difference was observed in the duration of surgery as several surgeons, including novices as well as experts, performed the surgeries.

## Discussion

The advantage of A-vit is that it does not include the posterior vitreous and can be performed by physicians who do not perform vitrectomies. Thus, although no significant difference was observed in the duration of surgery, A-vit is less invasive and can be completed in a relatively short time. In addition, the risk of eyeball compression and retinal detachment associated with peripheral vitrectomy can be reduced.

One of the advantages of Vit is that the fundus can be visualized clearly. Since the peripheral area can be visualized easily, appropriate measures can be taken if retinal tears or detachments are observed. The safety of vitreous surgery has improved in recent years with the introduction of the high-speed vitreous cutter and micro incision vitreous surgery [9]. Furthermore, the use of wide-angle observation systems during vitrectomy has reduced the invasiveness of macular surgery [10–12]. It is possible to spontaneously close the scleral wound without suturing by deliberately leaving the vitreous at the periphery to reduce retinal contact during the procedure [13, 14]. However, the low postoperative intraocular pressure was as significant in the PPV group as that in the A-vit group, which was thought to be a result of non-suturing of the invasion at the end of surgery and the effect of intraoperative eye compression on the ciliary body.

The vitreous was maintained, and no peripheral eye compression was observed in the A-vit group. Thus, the intraocular aqueous humor circulation was closer to the original status, suggesting that iris capture may be less likely to occur. There is a risk of fragility of the ciliary zonule due to peripheral compression. In contrast, if no posterior vitreous detachment was observed during vitrectomy in the PPV group, it was created artificially, which may result in the formation of a retinal tear or detachment. This step should be performed by a vitreous surgeon as there can be a complication that can affect visual prognosis if the required treatment is not administered properly. Patients with IOL dislocation usually visit the hospital or clinic where they underwent cataract surgery. These patients must be referred to another hospital if the vitreous surgeon is unavailable.

Significant differences were observed between the preoperative and postoperative best-corrected visual acuity (logMAR). However, no significant difference was observed between the groups. The extent of vitrectomy showed no significant difference in terms of the incidence of intraoperative complications. A significant difference was observed between the PPV group (*p* = 0.00041) in terms of the causes of intrascleral fixation after vitrectomy and scleral buckle, which may be attributed to the damage to the zonule during vitrectomy and scleral buckle. Thus, eyes with previous vitrectomy were considered to be risk factors for the IOL dropping into the vitreous cavity.

No difference was observed in terms of the visual prognosis or complications, except for low postoperative intraocular pressure, between the A-vit and PPV groups in the present study. However, the average follow-up period was short (approximately 2 years). Therefore, it is necessary to increase the number of patients and compare the complications in further studies with long-term follow-up.

As a future issue, we did not find any significant difference in the extent of vitreous resection in intravitreal scleral fusion; however, we believe that a detailed classification of cases, such as those requiring extensive vitreous removal or those in which multiple surgeries have been performed, or narrowing the number of surgeons may lead to a difference. In the future, we would like to increase the number of cases and examine long-term complications in consideration of this point.

## Conclusions

No significant difference in the postoperative visual acuity and the incidence of intraoperative and postoperative complications during intrascleral fixation according to the extent of vitrectomy were observed in the present study. The findings of the present study suggest that vitrectomy can be performed safely via the anterior approach alone during intrascleral fixation, depending upon the patient’s condition.

### Electronic supplementary material

Below is the link to the electronic supplementary material.

Supplementary Material 1

## Data Availability

Data sharing is not applicable to this article as no datasets were generated or analyzed during the current study.

## Abbreviations

A-vit group: Anterior vitrectomy group
ERM: Epiretinal membrane
IOL: Intraocular lens
LogMAR: Postoperative best-corrected visual acuity
LogMAR: Preoperative visual acuity
PCR: Posterior capsule breakage
PPV group: Pars plana vitrectomy group
